# Clysters of Pure Water

**Published:** 1854-04

**Authors:** 


*Clysters of Pure Water.*—Falck has made a series of experiments on the action of clysters of pure water, from which he obtained the following important practical results :

1st. Water clysters, to the amount of 330 grmms., have at any temperature between 0° and 40° R. (32° and 122° F.) a sanguipetal action, that is to say, they produce no evacuation, but the water becomes absorbed and enters the blood. If the water is of a temperature from 30° to 40° R., (100° to 122° F.) they excite a feeling of warmth, and if from 0° to 20° R. (32° to 77° F.) a feeling of cold, and according to the temperature act either as an astringent or a laxative.

2d. Water clysters, in which the amount of water introduced into the bowels is greater, say 660 grmms., have only within the temperature of 10° to 35° R., (55° to 110° F.) a sanguipetal action; at a higher or lower temperature they act as a purgative. According to their temperature they are either laxative or astringent. From these results, the following rules for their practical application are drawn:

1st. If water clysters are given with the intention of being absorbed, the volume must not be too great, and the temperature must approach as near as possible to that of the body, at best a temperature of 26° R. (90° F.)

2d. If the object be to produce evacuations, a large quantity of water must be used, and that temperature selected, according as a laxative or astringent is desired, either under 10° R. (55° F.) or above 35° F.) —*Translated from Vierordt's Archiv. f. physiol.*

